# Three-Dimensional Evaluation of the Cytotoxicity and Antibacterial Properties of Alpha Lipoic Acid-Capped Silver Nanoparticle Constructs for Oral Applications

**DOI:** 10.3390/nano13040705

**Published:** 2023-02-12

**Authors:** Dina Abdelmoneim, Gemma Porter, Warwick Duncan, Khoon Lim, Richard Easingwood, Tim Woodfield, Dawn Coates

**Affiliations:** 1 Sir John Walsh Research Institute, Faculty of Dentistry, University of Otago, Dunedin 9010, New Zealand; 2 Christchurch Regenerative Medicine and Tissue Engineering (CReaTE) Group, Department of Orthopaedic Surgery and Musculoskeletal Medicine, University of Otago, Christchurch 8011, New Zealand; 3 Otago Micro and Nanoscale Imaging, Department of Anatomy, University of Otago, Dunedin 9016, New Zealand

**Keywords:** silver nanoparticles, hydrogels, GelMA, antibacterial, scaffolds

## Abstract

There is a need to develop bifunctional scaffolds that provide antibacterial protection while encouraging host cell attachment/proliferation. This study evaluates HyStem^®^-C, and photo-cross-linked GelMA hydrogels for encapsulation and stabilisation of silver nanoparticles (AgNPs). We studied the behaviour of AgNPs and matrix interactions within both hydrogel systems. The cell viability of encapsulated human gingival fibroblasts (HGFs) was determined by Prestoblue^®^ assay and live/dead staining. The release of AgNPs was monitored by inductively coupled plasma–mass spectroscopy. The antibacterial properties of the GelMA-AgNP constructs were determined using disc diffusion. Even distribution of AgNPs in GelMA induced a significant decrease in cell viability (*p* < 0.0001), whereas AgNP aggregates did not induce cytotoxicity in HyStem^®^-C. AgNPs doses ≥ 0.5 µg/mL in GelMA were significantly toxic to the HGFs (*p* < 0.0001). The release of AgNPs from GelMA after 48 h was 20% *w*/*w* for 0.1 µg/mL and 51% for 100 µg/mL of AgNPs. At ≥5 µg/mL, a significant intra-construct bactericidal effect was observed. The disc diffusion assay shows that GelMA-incorporated AgNPs were found to be effective against both *Escherichia coli* and *Staphylococcus aureus* at 50 and 100 µg/mL, respectively. Visible photo-cross-linked GelMA stably incorporated AgNPs to provide an antimicrobial regenerative construct for oral applications.

## 1. Introduction

Infections are a common complication of biomaterial placement and often associated with high rates of morbidity due to surgical revision and failure. Thus, a need to fabricate biomaterials with antibacterial properties to hinder the formation of pathogenic biofilms is required. One of the most common modification methods is the incorporation of an antibiotic; however, over recent years, the rate of biomaterial failure caused by antibiotic-resistant bacteria has significantly increased [1]. Silver is well known to be a potent antimicrobial agent alterative to antibiotics. In addition to its broad antibacterial effects, silver has other properties such as biocompatibility, sustained release, and low bacterial resistance [2]. With recent advances in nanotechnology, silver nanoparticles (AgNPs) demonstrated superior properties to traditional bulk silver [3,4].

AgNPs have been studied in conjunction with wound dressings, prosthetic device coatings, topical agents, mouth washes and some dental restorative materials [5,6]. The increase in applications of AgNPs in the biomedical field has been attributed to its antibacterial, anti-fungal [7], anti-platelet [8], anti-proliferative [9], anti-inflammatory activity [10], and use as a cancer therapy [11]. Despite the widespread use of AgNPs, there is a shortage of information relating to their biological effects on both bacterial and human cells. Some studies linked the cytotoxicity of AgNPs to several possible mechanisms, including the disruption of the cell membrane, oxidative stress, protein or DNA damage, generation of reactive oxygen species, and induction of apoptotic cell death [12]. The characteristics of the AgNPs including size, shape, surface modification, and use of a capping agent, play a crucial role in determining the toxicity of the AgNPs [6]. It has also been found that the capping agent plays a role in controlling the size of the AgNPs and their stability over time [13].

Alpha lipoic-acid is five-membered cyclic disulphide tailing a short hydrocarbon chain on one end and a (COO-) carboxylic group on the other, known to be both antioxidant and anti-inflammatory [14]. While other silver formulations have clinical limitations due to negative effects on eukaryotic cell lines, the COO- group shows reduced cytotoxicity [15]. Our group has previously examined the utility of alpha lipoic acid capped AgNPs for intra-oral applications by investigating their cytotoxicity on human gingival fibroblasts (HGFs) and antibacterial properties on a broad range of oral pathogens in a traditional 2D culture [14]. The traditional 2D system involves growing cells as a monolayer on a flat plastic platform, which limits fundamental cellular behaviours such as cell-to-cell interactions and cell-to-matrix signalling [16]. Fibroblasts suspended in 3D systems in contrast to 2D systems exhibit bipolar morphology, and their cytoskeletal organization is less stringent, frequently lacking discrete focal contacts and stress fibers [17,18]. Additionally, 2D systems have been associated with changes in gene expression, which often leads to misinterpretation of cellular behaviour and functioning [19]. Therefore, there is a considerable shift to study cellular behaviour in models that better mimic the in vivo biology.

Our previous study on 2D culture showed concentration-dependent cytotoxicity where HGFs treated with ≤5 μg/mL had no adverse effects on cell viability, whereas concentrations ≥12.5 μg/mL caused a significant decrease in cell viability [14]. AgNPs had inhibitory minimal concentrations ranging (MICs) between 2.5 μg/mL for *Escherichia coli* DH5α (*E*. *coli*) and 12.5 μg/mL for *Staphylococcus aureus* (*S. aureus*). This study also reported the formation of AgNP aggregates in tissue culture media leading to poor distribution and sedimentation [14].This instability in cell culture media may influence the determination of the accurate antibacterial dosages.

Polymeric hydrogels containing AgNPs have attracted great attention for application in the biomedical field, particularly wound dressings and regenerative biomaterials. Gelatin methacryloyl, known as GelMA, is a popular natural polymer-based hydrogel composed of gelatin polymer chains chemically functionalised by methacryloyl (MA) groups [20]. Gelatin is derived from the hydrolysis of collagen, which is the major component of the extracellular matrix (ECM). Besides being biocompatible and biodegradable, it is a popular 3D scaffold owing to the arginine–glycine–aspartic acid sequences that promote cell attachment, as well as the matrix metalloproteinase sequence that encourages cell proliferation and remodelling [21,22]. The MA groups on GelMA can further undergo cross-linking in the presence of a recently developed photo-initiating system consisting of a ruthenium complex (Ru) and sodium persulfate (SPS), as well as visible light (wavelength = 400–450 nm) [23]. This approach is proven to be safer with mammalian cells when compared with the traditional UV cross-linking [23,24].

In the present paper, we describe the integration of AgNPs into both GelMA and HyStem^®^-C, representing different hydrogel systems. HyStem^®^-C is composed of hyaluronic acid (HA), which is another major constituent of the ECM. According to the manufacturer, denatured collagen was further added this product to enhance cellular adhesion and attachment. Modified forms of HA have been widely studied as wound healing biomaterials and in drug delivery systems [25,26,27]. HA is a glycosaminoglycan known to be biocompatible and biodegradable, and can perform important ECM functions such as regulating cell adhesion and migration, while mediating proliferation and differentiation [28].

Within this context, this research aimed to study the cytotoxicity and antibacterial properties of alpha-lipoic-acid-capped AgNPs by encapsulating both human and bacterial cells in 3D hydrogel scaffolds containing AgNPs in order to (1) prevent AgNP sedimentation and aggregation, (2) to maintain proper AgNP particle distribution and retention of functionality throughout the experiment, and (3) to provide physiological similarities to the native ECM. This study optimised the constructs size and consistency for both hydrogel systems, examined the AgNP/hydrogel interactions and determined both cytotoxicity and antibacterial properties of alpha-lipoic silver nanoparticles in GelMA.

## 2. Methods

### 2.1. Alpha-Lipoic-Acid-Capped Silver Nanoparticles Preparation and Characterisation

AgNPs were prepared and characterised as previously described by Cotton et al., 2019 [14]. Concentration was quantified using inductively coupled plasma-mass spectrometry (ICP-MS, Agilent 7500 ce instrument, Agilent Technologies; Santa Clara, CA, USA), by adding 4 mL of concentrated HNO_3_ in a Teflon digestion vessel (Cat. No. 010-500-264; SPS Science, Sherbrooke, QC, Canada), followed by gentle heating. The samples were digested at 95 °C for 1 h. During the digestion process, the sample’s volume was reduced to ∼0.2 mL, which was made up again to 3 mL using deionised water. Transmission electron microscopy (TEM) images was obtained using a Philips CM100 BioTWIN TEM (Philips/FEI Corporation; Eindhoven, Holland) equipped with a LaB6 emitter fitted with a MegaView III Olympus digital camera. The samples were prepared by depositing 10 μL of alpha-lipoic-acid-capped AgNPs onto plasma-glowed, carbon-coated (400 mesh) copper grids. After 60 s, the excess was blotted with filter paper and the sample was left to air dry before viewing. The particle size diameter was measured manually for 100 particles using ImageJ software version 2023, U. S. National Institutes of Health, Bethesda, MD, USA.

### 2.2. Optimisation and Characterisation of the Hydrogel Constructs

#### 2.2.1. HyStem^®^-C Reconstitution and Sample Preparation

HyStem^®^-C (Cat. No. HYSC020-1KT; Sigma-Aldrich, Auckland, New Zealand), was used as per manufacturer instructions. Briefly, the hydrogel components were brought to RT and both Gylcosil and Gelin were reconstituted with 1 mL of degassed water (DG-H_2_O). Different hydrogel consistencies (stiff, standard, and soft) were prepared by reconstituting the Extralink with different volumes of the DG-H_2_O, as per Table S1A in the Supplementary Materials, and then gently inverted several times to mix. Gelin and Glycosil were incubated for 30 min at 37 °C, 5% CO_2_, then placed horizontally on a shaker for 2 h (60 cycles per min), until fully dissolved. Within 2 h of reconstituting the solutions with the degassed water, Glycosil and Gelin were mixed in a ratio of 1:1, and slowly pipetted up and down to avoid aeration. The Glycosil/Gelin mix was then placed on a shaker for a further 30 min. The cross-linker was added, and the constructs pipetted directly into a well plate and left undisturbed for 20 min to allow for gelation. AgNPs were added at a concentration of 200 μg/mL or control carrier (deionised water, pH 10) to maintain a constant volume.

#### 2.2.2. GelMA Synthesis

GelMA was synthesised as previously described by Lim et al., 2019 [23]. Briefly, gelatin (porcine skin, type A, 300 g Bloom strength, Cat. No. G1890; Sigma Aldrich, St. Louis, MO, USA) was dissolved in PBS at 10 wt%, with 0.6 g of methacrylic anhydride (Cat. No. 276685; Sigma Aldrich, St. Louis, MO, USA) per gram of gelatin added to the solution and left to react for 1 h at 50 °C under constant stirring. The obtained solution was then filtered through a 0.22 µm sterile filter, lyophilized under sterile conditions and stored until use. Prior to use, different stock solutions were prepared as per Table S2A in the Supplementary Materials, by dissolving the lyophilized GelMA in PBS and incubating at 37 °C overnight.

#### 2.2.3. GelMA Reconstitution and Sample Preparation

GelMA constructs were prepared by adding 50% *w*/*w* of GelMA stock solution, 48% PBS, 1% of 50 mM tris(2,2-bipyridyl) dichlororuthenium(II) hexahydrate (Ru; Cat. No. 544981; Sigma-Aldrich, Auckland, New Zealand), and 1% of 500 mM sodium persulphate (SPS; Cat. No. 216232; Sigma-Aldrich, Auckland, New Zealand). Stock solutions of 50 mM Ru (MW = 748.63 g/mol) and 500 mM SPS (MW = 238.1 g/mol) were made in PBS. All solutions were sterilized with a 0.22 μm filter prior to use. Different gel consistencies (stiff, standard, and soft) were obtained by preparing different concentrations of the stock GelMA solution as per Table S2A in the Supplementary Materials. In the GelMA-AgNP constructs, the volume of AgNPs to be used was deduced from the total amount of PBS added. Constructs were pipetted into well plates and photo cross-linked for 3 min using visible light (Wavelength = 400–450 nm) at 30 mW/cm^2^.

#### 2.2.4. Cell Culture of Primary Human Gingival Fibroblasts

Primary human gingival fibroblasts (HGFs) were obtained with consent from a female patient undergoing routine crown-lengthening surgery at the Faculty of Dentistry, University of Otago. All experiments were performed in accordance with the guidelines of the National Ethics Advisory Committee, New Zealand, and approved by the Human Ethics Committee, University of Otago, reference number H17/112. The HGFs were grown in Dulbecco’s Modified Eagle Medium (DMEM; Cat. No. 10569010; Life Technologies New Zealand limited, Auckland, New Zealand) supplemented with 10% Fetal Bovine Serum (FBS; Cat. No. 10091148; Life Technologies New Zealand Limited, Auckland, New Zealand), 1% Antibiotic-Antimycotic (Anti-Anti; Cat. No. 15240062; Life Technologies New Zealand Limited, Auckland, New Zealand), 0.5% Gentamicin (Cat. No. 15710064; Life Technologies New Zealand Limited, Auckland, New Zealand). Cells were centrifuged at 1000× *g* for 5 min and the cell pellet resuspended at 5 million cells/mL of hydrogel as described below.

#### 2.2.5. Cell Encapsulation in HyStem^®^-C and GelMA Constructs

Encapsulation of HGF cells within the HyStem^®^-C constructs were conducted by resuspending the cell pellet at a ratio of 4:1 Glycosil/Gelin mix to Extralink (cross-linker). Constructs of 10 and 25 μL at the three different consistencies of each hydrogel were pipetted into a 96-well plate and incubated at 37 °C and 5% CO_2_ for 20 min to allow for gelation before adding the media. After gelation, 400 μL of media was gently added at the side of the well to avoid disturbing the construct (*n* = 3). GelMA/HGF constructs were produced by resuspending the cell pellet in GelMA/PBS and mixing gently, followed by the addition of the cross-linkers Ru and SPS. Constructs of 10 and 25 μL in size were pipetted into a 96-well plate and photo cross-linked for 3 min using visible light (Wavelength = 400–450 nm) at 30 mW/cm^2^ (*n* = 3). After cross-linking, 400 μL of media was added gently at the side of the well and the constructs were incubated at 37 °C, 5% CO_2_. Three cell-free controls of each condition were prepared.

#### 2.2.6. Metabolic Activity of HGFs within the Hydrogel Constructs

The metabolic activity of cells in the two hydrogels at construct sizes of 10 μL and 25 μL (*n* = 3) with stiff HyStem^®^-C and standard GelMA was determined by incorporating AgNPs at a toxic dose of 200 μg/mL with 5 million HGF cells/mL, as described above. The samples were incubated at 37 °C, 5% CO_2_, for 20 h, then 40 μL of Prestoblue^®^ cell viability regent (Cat. No. G8080; Promega, In Vitro Technologies, Beverly Hills, CA, USA) was added to each well. The well plates were incubated again at 37 °C, 5% CO_2_, and fluorescent measurements recorded the 24 and 48 h time points. Metabolic activity was measured by transferring 50 μL of solution to a 96-well plate, and fluorescence measurements were undertaken using a Synergy 2 Multi-mode microplate reader with an excitation/emission of 535/615 nm.

#### 2.2.7. HGF Cell Viability by Live/Dead Staining

Following the cytotoxicity experiments, the same constructs were labelled using cell viability imaging (Cat. No. R37610; Life Technologies, New Zealand limited, Auckland, New Zealand). Constructs were washed with PBS (×3), and 15 μL of both NucBlue^®^ and propidium iodide added to each well plate in 400 μL of fresh DMEM/10% FBS. The well plate was incubated for 45 min and then washed (×3) with PBS, prior to being observed under a Nikon-inverted confocal microscopy (Japan). Representative images were used for counting live/dead cells using ImageJ software.

### 2.3. Silver Nanoparticles and their Interaction with Hydrogels

#### 2.3.1. Morphology and Elemental Analysis

Samples were prepared as previously outlined and stored overnight at −80 °C before freeze drying for 24 h. Scanning electron microscopy with energy dispersive spectroscopy (SEM-EDS) analysis of the carbon-coated stiff HyStem^®^-C and standard GelMA with and without 100 μg/mL of AgNPs was performed using a JEOL JEM-6700F (Tokyo, Japan). EDS spectra were obtained from ten randomly chosen areas per sample. Five areas of approximately 5 mm × 5 mm were selected from the electrodense (white) areas representing smaller white areas of NaCl crystals or AgNPs, and five were selected from the greyish-black areas representing the matrix.

#### 2.3.2. TEM Imaging of Hydrogels Constructs with and without AgNPs

TEM imaging was performed on standard 10 μL GelMA constructs and 10 μL HyStem^®^-C constructs containing 0 or 200 μg/mL AgNPs. Prior to imaging, the media was removed from the wells and replaced with a 100 μL of blue food dye to ensure the gels were visible during processing, particularly the AgNP-free HyStem^®^-C constructs, which were translucent. After 20 min, the food dye was removed, and the constructs were washed with PBS and then fixed with 2.5% glutaraldehyde in 0.1 M sodium cacodylate for 1 h. Constructs were then washed twice for 5 min in 0.1 M sodium cacodylate followed by a third wash in double distilled water for 5 min. The gels were carefully removed from the wells with a spatula and transferred to 1 mL of distilled water in 1.5 mL tubes. Water was removed with a plastic transfer pipette, with a 150-mesh copper grid on the tip, to aid in the retention of the specimens. The specimens were exposed to serial dehydration using ethanol at 70%, 80%, 95%, and 100% for 5 min each, and then infiltrated with a 50:50 mixture of low viscosity Spurr’s resin and 100% ethanol for 30 min. This was followed by a second infiltration with 100% Spurr’s resin for 1 h. The old resin was then replaced with fresh resin and allowed to infiltrate for another 30 min. Finally, the constructs were removed and placed into silicone moulds, which were filled with resin, covered and cured for 48 h at 60 °C. The cured resin blocks were removed from the silicone moulds and semi-thin sections (~3 µm thick) were prepared. The sections were stained with methyl blue to verify the presence of gel before ultrathin sections of 80 nm were prepared and stained with 0.5% uranyl acetate and 3% lead citrate for contrast (LKB Ultrostain or Leica EM Stain machine). Unstained sections were used for better viewing of AgNPs. The sections were mounted onto formvar-coated copper slot grids and viewed under JEOL JEM-2200FS transmission electron microscope.

#### 2.3.3. Fourier Transform Infrared-Attenuated Total Reflection (FTIR-ATR)

Samples were prepared as previously outlined and pipetted into 6 mm diameter silicone moulds, then stored overnight at −80 °C before freeze-drying for 24 h. Spectra of stiff HyStem^®^-C and standard GelMA with and without 100 μg/mL of AgNPs were obtained using an FTIR-ATR spectrometer (Alpha II, Bruker, Germany). For each condition (*n* = 2), 100 scans were acquired in the 4000–400 cm^−1^ range, with a resolution of 4 cm^−1^.

### 2.4. GelMA as a 3D Model for Cytotoxicity

#### 2.4.1. AgNP Cytotoxicity on Encapsulated HGFs in Standard GelMA Constructs

To determine the concentration of AgNPs producing cytotoxic effects, 10 μL constructs of standard GelMA were prepared with and without 5 million HGF cells/mL, as described. AgNPs were incorporated at 0, 0.1, 0.5, 1, 5, 10, 50, and 100 μg/mL (*n* = 3), and a cell viability assay was preformed and measured as described in Section 2.2.6.

#### 2.4.2. Silver Nanoparticles Release from GelMA Constructs

ICP-MS was used to determine the concentration of AgNPs released from the GelMA constructs after 48 h of in vitro incubation in 400 μL of DMEM/10% FBS. Constructs of standard consistency of GelMA (5 wt%) at 10 μL in size were prepared with and without the encapsulation of 5 million HGFs/mL. AgNPs were incorporated at the following concentrations: 0, 0.1, 0.5, 1, 5, 10, 12.5, 25, 50, 100 and 200 μg/mL (*n* = 3). After 48 h incubation at 37 °C/5% CO_2_, 250 μL of the media supernatant was transferred to a Teflon digestion vessel and conducted using ICP-MS. A standard curve was generated by preparing media containing AgNPs at 0, 0.0002, 0.002, 0.02, 0.2 and 2 μg/mL.

#### 2.4.3. TEM Imaging of GelMA Constructs

TEM imaging was performed on 10 μL GelMA constructs encapsulating 5 million HGFs/mL and containing 0, 0.1, 100, and 200 μg/mL of AgNPs (Section 2.3.2). The average particle size diameter of the AgNPs incorporated in GelMA was also determined by measuring 50 nanoparticles per image (*n* = 3) using ImageJ software.

### 2.5. Antibacterial Properties of AgNPs Encapsulated in GelMA

#### 2.5.1. Bacterial Culture

*Escherichia coli* DH5α (*E*. *coli*), and *Staphylococcus aureus* Oxford NCTC6571 (*S*. *aureus*) were obtained from the University of Otago culture collection, Dental School and sub-cultured on Tryptic Soy agar plates (Cat. No. 1335; Fort Richard, Mt. Wellington, New Zealand) with incubation at 37 °C/5% CO_2_ for 24 h. Sixteen hours prior to experiments, the species were inoculated into 10 mL of tryptic soy broth (TSB; Cat. No. 211825; Difco Laboratories, Detroit, MI, USA) and incubated at 37 °C/5% CO_2_.

#### 2.5.2. Bacterial Encapsulation in GelMA

Prior to the encapsulation of *E*. *coli* and *S*. *aureus* into GelMA, a bacterial optical density (OD) of 0.01 per ml of hydrogel was calculated. The amount of broth was subtracted from the total volume of PBS used in the GelMA construct. Then, 10 μL constructs were cross-linked at 450 nm for 3 min in a 48-well plate and incubated at 37 °C/5% CO_2_ for 20 h.

#### 2.5.3. Minimal Inhibitory Concentration (MICs) and Minimal Bactericidal Concentration (MBCs) in 3D Culture

To determine the MICs of AgNPs/GelMA, *E. coli* and S. *aureus* were encapsulated in 10 μL constructs of GelMA with 0, 0.1, 0.5, 1, 5, 10, 50, and 100 μg/mL of AgNPs (*n* = 3) and no bacterium as a background control. The samples were pipetted into a 48-well plate, placed in a sealable plastic bag to maintain humidity, and incubated at 37 °C/5% CO_2_. After 20 h of incubation, 400 μL of broth with 40 μL of the Prestoblue^®^ regent were added, and the well plates were incubated for another 3 h before recording at the 24 h time point. Metabolic activity was measured by transferring 50 μL of solution from each well plate to a 96-well plate, and fluorescence measurements were undertaken using a Synergy 2 Multi-mode microplate reader with an excitation/emission of 535/615 nm. MICs was measured as the lowest concentration resulting in bacterial growth inhibition. MBCs was determined by spot-plating 10 μL of the broth onto TSA agar plates, followed by incubation for 24 h, and was recorded as the lowest concentration resulting in completely killing the bacteria.

#### 2.5.4. Antibacterial Properties of GelMA Incorporated AgNPs by Disc Diffusion Assay

The bacterial OD of *E*. *coli* and *S*. *aureus* was measured and adjusted to 0.1 OD. One ml of the adjusted broth was added to 19 mL of TBS and mixed slowly up and down before pouring in an empty sterile Petri dish, and then left to set. Using a biopsy punch, 3 holes measuring 5 mm in diameter were punched per plate. In total, 10 μL of the standard consistency (5 wt%) of un-cross-linked GelMA with the following concentrations of AgNPs, 0, 25, 50, 100, and 200 μg/mL, was pipetted directly inside the punched hole and cross-linked for 3 min. Afterwards, the plates were incubated for 24 h at 37 °C/5% CO_2_. Images were taken using a Canon camera and the inhibition zone diameters was measured using Image J software.

### 2.6. Statistical Analysis

The results were reported as the mean ± standard deviation. The statistical analysis of the results was performed using one-way ANOVA with post hoc analysis using GraphPad Prism. *p* ≤ 0.05 was considered to be statistically significant.

## 3. Results

### 3.1. Synthesis of Alpha-Lipoic-Acid-Capped Silver Nanoparticles

**Figure 1:**
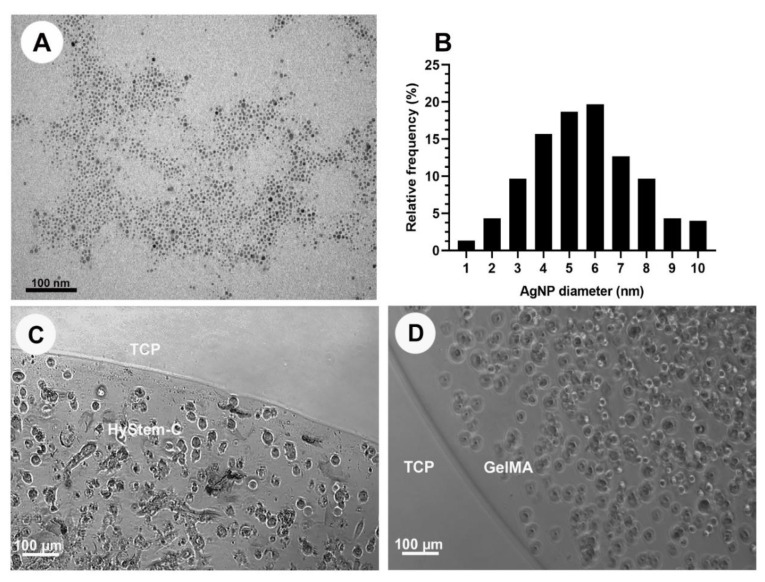
(**A**) Transmission microscope image of alpha-lipoic-acid-capped AgNPs. (**B**) Histogram showing the size distribution of the AgNPs used in the study. (**C**) Human gingival fibroblasts (HGF) encapsulated in stiff HyStem^®^-C loaded with 200 μg/mL AgNPs at 48 h. (**D**) HGF encapsulated in standard GelMA loaded with 200 μg/mL AgNPs at 48 h. TCP—tissue culture plastic.

After synthesis of the colloidal suspension of AgNPs, their appearance was dark yellow/brown in colour, which indicated stability, as well as good dispersion of the nanoparticles. The hydrodynamic size of AgNPs measured using DLS was determined to be 1–12 nm in size with a concentration ranging between 1800 and 2900 μg/mL. AgNPs were used within 14 days of being produced. TEM images showed spherical particles with an average diameter of 6 nm (Figure 1A,B).

### 3.2. Optimisation and Characterisation

**Figure 2:**
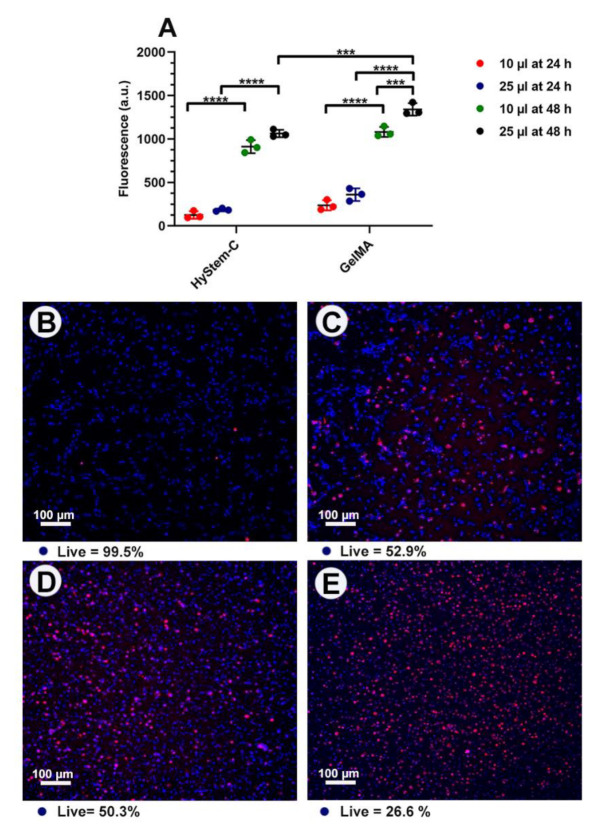
Top: (**A**) Graph showing cell viability of 25 and 10 µL construct sizes of both HyStem^®^-C and GelMAat 24 h and 48 h. Bottom: Confocal images of live/dead cells. Live cells—blue, dead cells—red. **** *p* < 0.0001, *** *p* < 0.001, (**B**) HyStem^®^-C 10 µL. (**C**) HyStem^®^-C 25 µL. (**D**) GelMA 10 µL. (**E**) GelMA 25 µL.

HGF cells were successfully encapsulated and maintained in HyStem^®^-C and GelMA with AgNPs (Figure 1C,D) and without AgNPs. The consistencies of hydrogels influenced the encapsulation process. Of the HyStem^®^-C consistencies tested, the stiff gels showed better retention of the AgNPs after 48 h (Figure S1, Supplementary Materials). The soft consistency, particularly for GelMA was rapidly degraded by the cells, and the cells were found to be growing on the tissue culture plastic and were therefore excluded from the experiment. Stiff gels (i.e., high viscosity) had a negative effect on the handling properties of GelMA and was associated with pipetting inaccuracies, leading to its exclusion. Thus, stiff HyStem^®^-C and a standard consistency of GelMA were used throughout the experiments. Similar values for HGF metabolic activity were seen with HyStem^®^-C and GelMA constructs of 25 µL and 10 µL at each time point (Figure 2A). At 24 h, there was no significant difference between the 25 µL and 10 µL constructs; however, there was a significant increase in metabolic activity with 25 µL GelMA at 48 h. Confocal microscopy showed a higher number of dead cells in the large-sized constructs (25 µL), compared with the 10 µL constructs, particularly in the middle of the construct (Figure 2D,E). Thus, the 10 µL construct was used as the preferred construct size in subsequent assays.

### 3.3. AgNPs-Hydrogel Matrix Interaction

**Figure 3:**
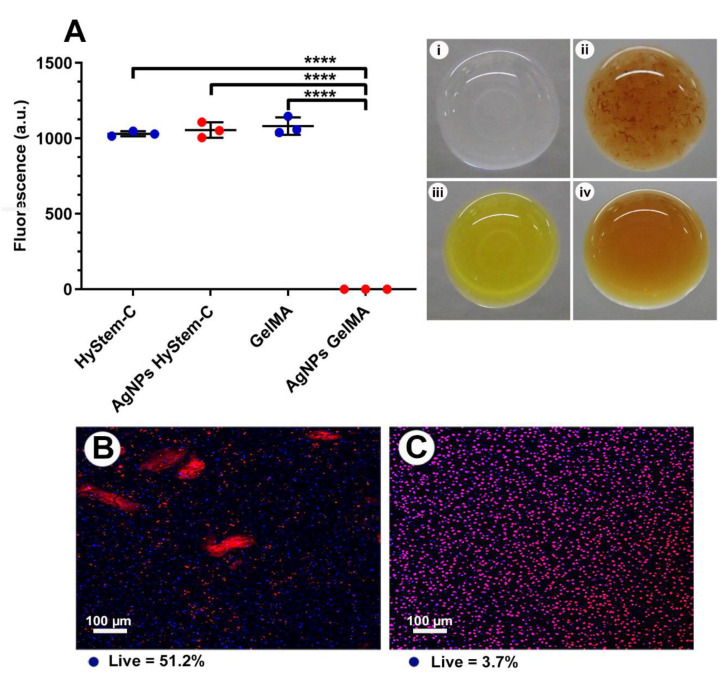
(**A**) Graph showing cell viability of HGFs encapsulated with 200 µg/mL AgNPs (toxic concentration), and controls. **** = *p* < 0.0001. Right, photographs of the two hydrogel systems; (**i**) HyStem^®^-C, (**ii**) HyStem^®^-C with AgNPs, (**iii**) GelMA, (**iv**) GelMA with AgNPs. Images in iii and iv were taken upon addition of AgNPs prior to cross-linking, aggregation of AgNPs in HyStem^®^-C but not GelMA can be seen. Confocal images of live/dead cells are shown in (**B**) HyStem^®^-C stiff and (**C**) GelMA standard containing 200 µg/mL AgNPs; live cells—blue, dead cells—red.

**Figure 4:**
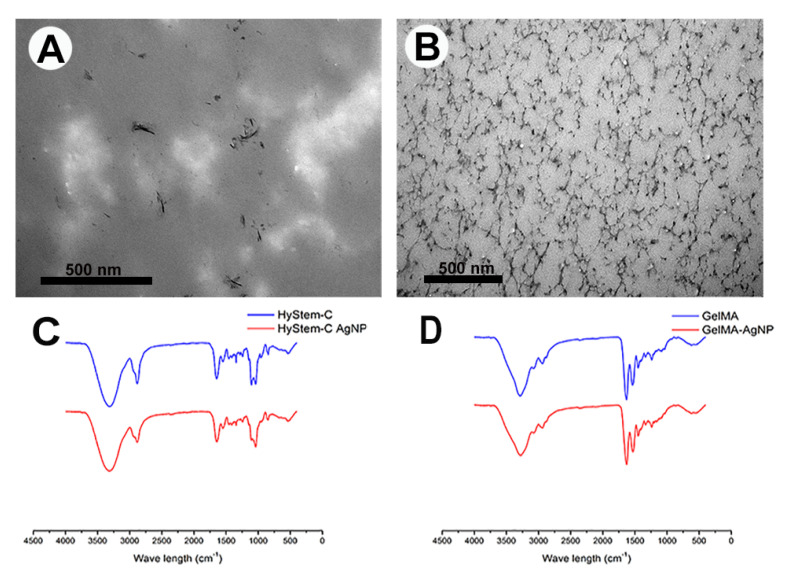
Top: TEM images stained with uranyl acetate and lead citrate showing distribution of 200 µg/mL AgNPs. (**A**) Small aggregates of AgNPs in HyStem^®^-C. (**B**) Well-dispersed AgNPs in GelMA. Bottom: FTIR-ATR Spectra of both hydrogels with and without 100 µg/mL AgNP. (**C**) HyStem^®^-C. (**D**) GelMA.

At the macroscopic level, aggregates of AgNPs were immediately observed upon addition to the hydrogel components of HyStem^®^-C, unlike GelMA where the AgNPs dissipated in the matrix and were not visible, indicating even dispersion of the NPs (Figure 3i–iv). As soon as AgNPs were added to GelMA matrix, the original yellow matrix changed to brown and then after cross linking with light for 3 min reversed again to yellow, which might indicate a chemical interaction (Figure 3iii,iv). Confocal imaging of 10 µL constructs containing 200 µg/mL AgNPs supported the idea that the AgNPs were entrapped and resulted in HGF cell death in GelMA, but not HyStem^®^-C (Figure 3B,C). TEM imaging revealed small silver aggregates in HyStem^®^-C that formed strands and clusters (Figure 4A). TEM of GelMA showed an even distribution of the AgNPs throughout the construct (Figure 4B). Thus, AgNPs at the same toxic concentration of 200 µg/mL did not result in cytotoxicity in HyStem-C; however, in GelMA with dispersed AgNPs, cytotoxicity was detected, suggesting a correlation between the retention and distribution of AgNP and their activity (Figure 3A).

FTIR-ATR spectra of the HyStem^®^-C and GelMA with and without AgNPs are presented in Figure 4C,D. The HyStem^®^-C spectra revealed typical stretching peaks of OH- groups appearing at 3315 cm^−1^. Additionally, typical absorption peaks of amides appeared at 1646 cm^−1^ (amide I), 1548 cm^−1^ (amide II) and 1241 cm^−1^ (amide III), which are the characteristic absorption peaks of C=O, N–H, and C–N, respectively [29]. The peak appearing at 1409 cm^−1^ is attributed to C=O stretching of the carboxyl groups within the HA [30]. Other significant peaks at 1105 cm^−1^, 1082 cm^−1^ and 1039 cm^−1^ are assigned to C-O ester association, C-C groups, and C-OH groups, respectively [31]. No new peaks, or significant shifts in wave numbers, were observed with the addition of AgNPs to the HyStem-C. Spectra derived from GelMA showed a broad peak at 3289 cm^−1^, attributed to the stretching of the hydrogen-bonded hydroxyl groups. The peaks at 1237 cm^−1^, 1537 cm^−1^ and 1630 cm^−1^ are associated with the C–N stretching of amide III, the N–H stretch of amide II, and C=O stretching of amide I, respectively. The spectrum also displayed the characteristic bands of N-H stretching at 3078 cm^−1^ (amide B), C-H stretching at 2937 cm^−1^, and 2878 cm^−1^ (amide A) and C-H deformation were also detected at 1444 cm^−1^. Other relevant peaks were identified at: 1161 cm^−1^ (symmetric C-O-C stretching), 1199 cm^−1^ (ν (O-C-O), and 1336 cm^−1^ (CH_2_). The spectra of the GelMA with AgNPs did not show any new peaks corresponding to the AgNPs, but a shift in certain peaks was identified; this occurred around the 3273 cm^−1^ region (corresponding to the -OH groups). In addition, amide II and amide I also shifted to lower wavelengths of −1529 cm^−1^, 1629 cm^−1^, respectively. (FTIR-ATR spectra of the HyStem^®^-C and GelMA with and without AgNPs with the relevant characteristic bands are presented in Figures S2 and S3, Supplementary Materials.)

**Figure 5:**
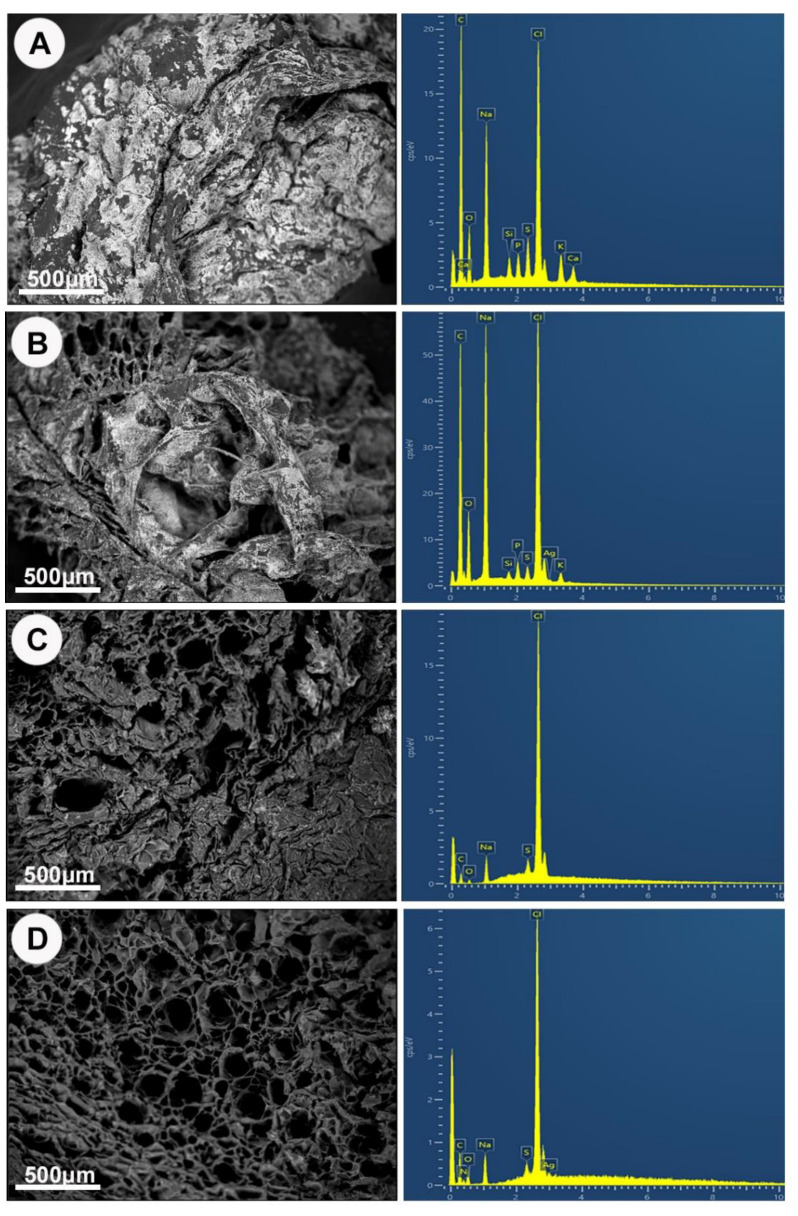
SEM images and respective EDS Spectra of both hydrogels with and without 100 μg/mL AgNP. (**A**) HyStem^®^-C. (**B**) HyStem^®^-C with AgNPs. (**C**) GelMA. (**D**) GelMA with AgNPs.

Figure 5 illustrates the SEM/EDS findings of HyStem^®^-C and GelMA constructs with and without AgNPs. A solid rough irregular surface morphology was evident with HyStem^®^-C hydrogels (Figure 5A,B), whereas the GelMA hydrogel (Figure 5C,D) exhibited irregular porous structures. Increased porosity was observed with the AgNPs containing GelMA compared with blank GelMA. To confirm the presence of AgNPs in the hydrogel matrix, EDS analysis was performed. The EDS spectrum of HyStem^®^-C and GelMA blank hydrogels (Figure 5A,C) were negative for an Ag element peak, whereas the EDS spectrum of AgNP-loaded hydrogels clearly showed a peak of Ag (Figure 5B,D). The spectra were acquired from randomly chosen but widely distributed areas. At a very high magnification (×5000), the Ag was detected everywhere within the matrix of the HyStem^®^-C AgNP-loaded samples, indicating aggregation in certain areas or oxidation of the AgNPs and loss of nanoparticle structure. Meanwhile, the AgNPs in GelMA appeared as scattered small triangulated white particles, consistent in size with the incorporated AgNPs, whereas in HyStem^®^-C with AgNPs, no particles were visible (Supplementary Materials: Figure S4). The SEM-EDS thus gave a distinct distribution pattern, and only in the GelMA + AgNPs constructs were discrete areas of Ag detected (Supplementary Materials: Table S3).

### 3.4. GelMA as a 3D Model

**Figure 6:**
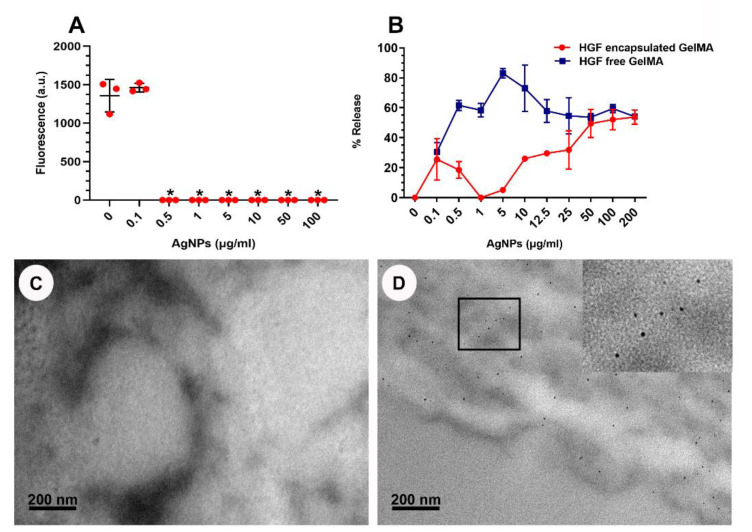
Top: (**A**) Graph showing cytotoxicity of AgNPs on human gingival fibroblasts after 48 h in GelMA. (**B**) Graph showing AgNPs release from GelMA with and without HGFs. (* *p* < 0.0001). Bottom: TEM images showing GelMA matrix without uranyl acetate and lead citrate staining. (**C**) GelMA matrix without AgNPS. (**D**) GelMA matrix with AgNPs at 200 µg/mL.

GelMA (10 µL) constructs made at a standard consistency were used as the preferred model due to an even AgNP distribution/retention and suitable handling properties. Concentrations ≥ 0.5 µg/mL of AgNPs encapsulated with HGFs showed significant cytotoxicity, with no cell growth (Figure 6A). An increase in AgNPs release of 20–60% *w*/*w* of AgNPs was observed from GelMA (10 µL) constructs without cells containing 0.1–1 µg/mL AgNPs (Figure 6B). However, GelMA constructs containing AgNPs at >1 µg/mL demonstrated a plateaued AgNP release of ~50% *w*/*w*, suggesting a maximum AgNPs concentration within the hydrogel and constant release upon incorporation of increasing AgNP concentrations. Constructs containing HGFs had a lower % release compared with the HGFs-free constructs. TEM imaging without uranyl acetate and lead citrate staining was conducted to retain the nanostructures. Figure 6C shows the background GelMA matrix (no AgNPs) while Figure 6D indicates dispersed 200 µg/mL AgNPs. The shape and size of the AgNPs in GelMA was analysed by TEM. The TEM images showed that the AgNPs were mainly spherical in shape and uniformly distributed in the hydrogel matrix without aggregation, with a size distribution ranging from 3 to 10 nm.

**Figure 7:**
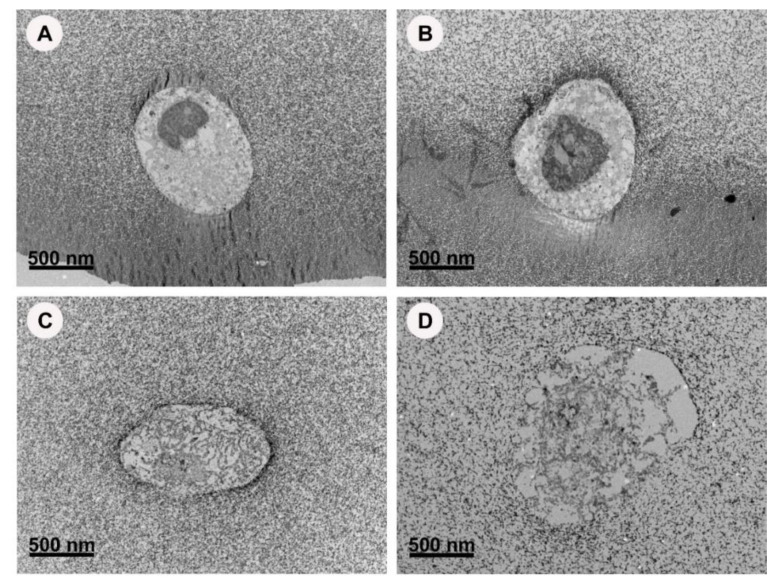
TEM images showing a human gingival fibroblasts cell encapsulated in GelMA containing different concentration of AgNPs. (**A**) Control, 0 µg/mL; (**B**) 0.1 µg/mL; (**C**) 100 µg/mL; (**D**) 200 µg/mL.

HGF encapsulated with 0 and 0.1 µg/mL AgNPs in 5%wt GelMA and (non-toxic concentration) was imaged using TEM (Figure 7A,B). The cell membrane appeared intact with a prominent nucleus. However, the internal structures were difficult to recognise or were lost. On the other hand, HGF encapsulated with toxic concentrations at 100 µg/mL had an interrupted cell membrane with the loss of the nuclear structure. At the highest concentration of 200 µg/mL, the cell membrane was completely deformed with the loss of the cell boundaries. The cellular content appeared dispersed within the gel content and no cell organelles could be identified.

### 3.5. Antibacterial Properties of GelMA Incorporated with AgNPs

**Figure 8:**
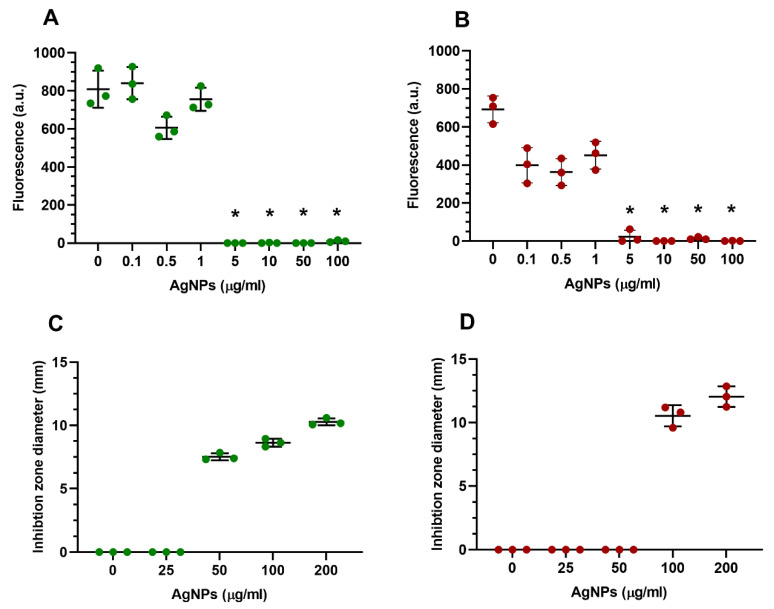
Top; Graphs the bacterial viability by Prestoblue^®^ at 24 h. (**A**) *E. coli*. (**B**) *S. aureus* * < 0.0001. Bottom: Graphs showing inhibition zones of *E. coli* and *S. aureus* of different concentrations of AgNPs encapsulated in GelMA constructs. (**C**) *E. coli*. (**D**) *S. aureus*.

The metabolic activity of *E*. *coli* and *S. aureus* encapsulated in 10 µL standard GelMA (Figure 8A,B) was investigated. At 24 h, ≥0.1 µg/mL concentrations slightly decreased the bacterial viability for both tested organisms; however, at ≥5 µg/mL, significant cytotoxicity was observed. At 48 h, the 10 µL plated broth did not show any bacterial growth at the concentrations of 5 µg/mL or higher, indicating complete killing of the tested bacterium (bactericidal activity).

The antimicrobial efficiency of the GelMA-AgNPs was tested using a modified disc diffusion method. Figure 8C,D shows the diameters of the clear zone (inhibition zone) of bacterial inhibition around the sample after a 24 h incubation of the agar plate at 37 °C. Diameters of inhibition zones varied with type of tested microorganism and silver concentration used in the constructs and ranged from 3 to 7 mm. Inhibition zones and, therefore, antimicrobial activity increased with increasing concentration of AgNPs encapsulated in the GelMA constructs. GelMA incorporated AgNPs were found to be effective against both *E. coli* and *S. aureus* at 50 and 100 µg/mL, respectively.

## 4. Discussion

In the present study, we studied alpha lipoic-acid-AgNPs incorporated in two hydrogel polymeric systems. The hydrogel composition and structure influenced the NPs behaviour and interaction. There was a correlation between cytotoxicity and the AgNP distribution. Even distribution of AgNPs in GelMA induced a significant decrease in cell viability; meanwhile, the same cytotoxic effect was not observed in the HyStem^®^-C at the same concentration. Superior distribution, retention, and release profiles of AgNPs from GelMA model were observed. At ≥5 µg/mL, a significant bactericidal effect against both *E*. *coli* and *S. aureus* at 50 and 100 µg/mL, respectively, was observed when GelMA-incorporated AgNP constructs were tested.

### 4.1. Significance of the Photo-Cross-Linked GelMA-AgNPs as Bifunctional Non-Antibiotic Scaffold

GelMA has emerged as a promising biomaterial due to its tailorable properties. The most used photo-initiator to cross-link GelMA is Irgacure 2959 that requires ultraviolet light (UV) which can potentially cause cellular DNA and tissue damage [32]. Additionally, UV light has been previously reported to have limited light penetration depth which restricts its in vivo applications. The GelMA described herein is cross-linked using visible light, making it feasible to fabricate large constructs for in situ applications, meanwhile, eliminating the biosafety issues associated with UV light.

The AgNPs used in this research have an average diameter size of 6 nm, It has been reported that smaller sized AgNPs possess greater antimicrobial properties in comparison to larger-sized AgNPs [33], due to the higher intracellular bioavailability of silver caused by better cell-particle contact and an increased release of silver ions. Additionally, the AgNPs synthesised for this research have additional functionality via capping with alpha lipoic-acid, which is known for its antioxidant and anti-inflammatory properties [34].

Current treatment options for infected wounds and bone defects are limited to the surgical debridement of affected tissue and high doses of antibiotics. The presence of biomaterials in situ further complicates the treatment plan. Once infection is established, biomaterials-mediated infections are resistant to treatment, even with high doses of antibiotics and tend to persist until the prosthetic device is removed [35]. It is also noteworthy that systemic antibiotics often fail to eradicate infections associated with biomaterials [36], possibly due to limited vascularisation within the constructs during the initial stages of healing. With the increasing problem of antibiotic resistant bacteria, the incorporation of the AgNPs offers an opportunity to develop an antibacterial non-antibiotic-based scaffold. The initial AgNP burst release from GelMA will prevent bacterial colonisation on the biomaterial surface and the GelMA matrix will provide a framework for host cell attachment and regeneration. The GelMA-AgNP constructs described here have potential application not only in dentistry for bone defects, but also as an antibacterial medicament in periodontal pockets, peri-implant pockets and may have potential for treatment of infected root canals, intra-oral and extra-oral ulcerations, along with orthopaedic applications.

### 4.2. Construct Design

Hydrogel’s properties, such as porosity, degradability, swelling and mechanical, are influenced by the nature and extent of cross-linking of the polymer during gelation, and are known to affect cell behaviour and function during tissue formation [37]. Different consistencies can be achieved by manipulating the degree of polymer concentrations and cross-linking between the polymeric chain of the hydrogel [38]. Increased cross-linking is accompanied by an increase in polymer stiffness, strength, viscosity, and a decreased porosity and degradation rate. It is known that traction stresses correlated to the stiffness of the hydrogel, and high stresses leads to nuclear distortion [39]. Our primary experiments showed that there was no significant effect on HGF viability among the 3 tested consistencies of HyStem^®^-C: stiff, standard, and soft (Supplementary Materials: Figure S1). The average pore size of the standard HyStem^®^-C is <15.9 nm [40], which is 2× bigger than the average size of the AgNPs measured using TEM. The stiff hydrogel better retained the encapsulated AgNPs making it the preferred option for formation of a bifunctional construct.

In total, 2.5%-soft GelMA construct consistency was rapidly degraded by the encapsulated cells at 48 h, and HGFs were detected growing on the tissue culture plastic. On the other hand, 10%-stiff GelMA had a high viscosity and was difficult to pipette making formation of gels challenging. Accordingly, both soft and stiff consistence were excluded. Celikkin. N et al. investigated the effect of the GelMA concentration on mesenchymal cells osteogenic differentiation by evaluating the extracellular matrix calcification. The results showed that 5% GelMA hydrogel has more potential in bone tissue engineering over 10% GelMA hydrogel in vitro [41]. In contrast, odontoblast-like cells showed a preference for stiffer GelMA gels of 10% and 15% in a study investigated fabrication of pre-vascularized, cell-laden hydrogel pulp-like tissue constructs. Another group encapsulated gold nanoparticles into a 10 wt% GelMA hydrogel by UV-induced chemical cross-linking, and they found that the alkaline phosphate activity, proliferation, viability, and osteogenic differentiation of adipose-derived stem cells were promoted [42]. Seemingly, individual cell lines will respond differently to the GelMA stiffness. Another critical aspect to consider is construct size, with denser and bulkier polymeric networks result in reduced diffusion of cell nutrients along with encapsulation stress [43], which could be a reason for the observed decrease in viability in the middle region of the 25 µL constructs. Therefore, 10 µL of construct size for stiff HyStem^®^-C and standard GelMA were chosen as the preferred models.

### 4.3. Polymeric Matrices and Interactions with AgNPs

It is difficult to control the shape, size, as well as stability of silver nanoparticles in solution since they undergo rapid oxidation and tend to form aggregates leading to a reduction in efficacy. Upon the addition of AgNPs to the HyStem^®^-C matrix, aggregates were formed macroscopically. Aggregation of AgNPs in HyStem^®^-C can be attributed to the affinity between the NPs to the denatured collagen in the hydrogels’ components. AgNPs have been used previously in cross-linking collagen due to its affinity to collagen fibres [44]. Porosity at a range of 60%–90% was reported to facilitate cell respiration and penetration, and maintain a moist environment on the wound’s surface [45], increased porosity in the AgNPs containing hydrogels indicates that the constructs are suitable for cellular metabolism and wound regeneration. The incorporation of the AgNPs in the two hydrogels was studied by EDS. Silver peaks were detected in all areas within the HyStem^®^-C AgNP loaded samples which might indicate the oxidation of the AgNP to Ag^+^ ions and/or the formation of large aggregates. AgNPs loaded in GelMA were only detected as small discrete white spots with a size consistent with the synthesised nanoparticles of approximately 6 nm. TEM images of AgNPs appeared as small clusters, clumps, and strands in the HyStem^®^-C samples and small-sized spherical particle in the GelMA. Similarly, the particles’ diameter found in GelMA matches the shape and size range of the originally characterised AgNPs pre-encapsulation. Similar results were reported where TEM images revealed that the 5 nm AgNP sizes were negligibly different before the AgNPs had been added to a porcine derived hydrogel; however, aggregation was detected with a bigger particle size of 50 nm [46].

Colloidal silver nanoparticles release silver ions at the surface of the metal which is considered the main source of its antimicrobial activities [47], making it a reservoir for the release of Ag+ ions, thus extending their antimicrobial shelf-life. Meanwhile, free silver ions are very reactive, when in the body they quickly combine with salts to form a less soluble compound such as silver chloride [48]. It can be concluded that for the greatest effectiveness, products should contain most of its silver content in the form of nanoparticles to retain the controlled release of Ag+ ions and thus antimicrobial activity.

The examined FTIR-ATR spectra revealed that HyStem^®^-C alone and with AgNPs displayed similar absorption peak shapes, and position indicating that adding AgNPs to the HyStem^®^-C does not produce structural changes as previously reported in literature with other hydrogels [49]. FTIR-ATR of GelMA, compared to GelMA-AgNPs revealed slight shifts in certain peaks associated with Amide I and II, these changes suggest the formation of electrostatic attractions and intermolecular interactions among the hydrogel components [50]. It appears that the incorporation of silver nanoparticles inside the GelMA matrix ensured sufficient stabilization of the particles size and form with controlled release of silver nanoparticles. The results also suggests that there might be a possible chemical interaction/binding between the GelMA components and the AgNPs, further investigations are be needed for confirmation.

### 4.4. Cytotoxicity

Cell viability assay did not show any statistical difference between these two different hydrogel models; however, live/dead stains showed a higher percentage of viable cells in the 10 µL of construct size for stiff HyStem^®^-C with a cytotoxic AgNP dose of 200 µg/mL (Figure 3). The addition of the same cytotoxic dose induced significant cell death inside the GelMA model. The hydrogel composition can clearly influence the AgNPs interactions, hence this can lead to misinterpretation of toxicity profiles. In the light of the above, 10 µL of standard GelMA consistency was chosen as the preferred model to test the cytotoxicity of AgNPs in a 3D model. AgNPs doses ≥ 0.5 µg/mL in GelMA were significantly toxic (*p* < 0.0001). The values obtained from our cytotoxicity experiment are considerably higher than values commonly reported in the literature [51,52], and previously reported by us for the same AgNPs on both fibroblast and osteoblast tested in 2D cell culture model [14]. Riberio et al. reported similar findings when AgNPs were incorporated in silk hydrogels, suggesting that silver nanoparticles beyond a concentration of 0.5% may be toxic [53]. The small size of the NPs used in this study along with dispersal within the matrix is the most likely reason for the increased cytotoxic effects, as seen in the TEM images. The TEM images also showed uptake of the AgNPs particles by the HGF which explains why there was a decrease in silver release when the cells were encapsulated along with AgNPs in the hydrogels. In our previous study, we also found that AgNPs were endocytosed within lysosomes of bone cells [54], we also observed the adsorption of the AgNPs on the remnants of the dead bacterium [14].

### 4.5. Mechanism of Action

The exact mechanism of the antibacterial action and cytotoxicity of AgNPs is not fully elucidated. Various theories have been reported regarding how AgNPs exert its toxicity action. It was proposed that AgNPs can penetrate the cell, causing cellular structure damage, thus leading to disruption of cell function, and cell death. Another theory states that the cell death is mediated by the induction of reactive oxygen species (ROS) [55]. ROS can oxidatively modify nucleic acids, proteins, lipids, and other cellular components, which results in DNA damage, disturbance of enzyme functions, alteration of membrane fluidity, and bacterial death [56]. In this study, we found that encapsulation of the AgNPs with the HGFs at toxic concentration led to the distortion of the cellular structures, disintegration of the cell membrane, cytoplasm leakage, and collapse of the cell at high concentrations. Similar effects were reported in our previous work [54], when osteoblast cells were treated with even lower concentrations of AgNPs at 5 µg/mL.

### 4.6. Antimicrobial Activity

The disc diffusion assay shows that GelMA-incorporated AgNPs were found to be effective against both *E. coli (Gram-negative bacteria)* and *S. aureus (Gram-positive bacteria)* at 50 and 100 µg/mL, respectively. Gram-negative bacteria were previously reported to be more susceptible to AgNPs than Gram-positive bacteria, owing to the difference in the organization of the peptidoglycan. The lower liability of Gram-positive bacteria can be attributed to the fact that the amount of peptidoglycan is comparatively more in Gram-positive than Gram-negative bacteria with a thinner cell wall of 3 nm compared with 30 nm [57]. The characteristics of the Gram-positive cell wall leave silver ions stuck onto the cell wall, preventing its action and renders bacterium comparatively more resistant [58]. In addition, Gram-negative bacteria contain negative charge lipopolysaccharides in the cell membrane which promotes the adhesion of AgNPs, making the bacteria more susceptible [59]. To conclude, these structural differences explain why Gram-positive *S. aureus* are less inhibited and Gram-negative *E. coli* shows substantial inhibition even at lower AgNPs concentrations.

Panacek et al. reported antimicrobial or MIC values of 50 µg/mL for *S. aureus* and 100 µg/mL for *E.coli*, when AgNPs of 10 nm were encapsulated in a methylcellulose hydrogel [60]. While ≥0.5 µg/mL doses of AgNPs induced significant cytotoxicity, ≥5 µg/mL were required to induce bactericidal effect for both tested bacteria. In the 3D model presented here, the mammalian cells were more susceptible to the AgNPs toxicity effect compared with the bacterial cells. This observation might be due to the small size of the bacterial cells (0.5–2.0 µm) compared with the mammalian cells (10–15 µm), enabling their escape through the porous hydrogel matrix. As such, the release profile and direct interactions of the GelMA-AgNP constructs are critical when tested in vivo. These constructs may provide good antimicrobial protection in the early phases of healing where cells are still infiltrating into the regenerative construct.

## 5. Conclusions

We found that the composition of hydrogel can greatly influence the cytotoxicity profile interpretation of AgNPs. Photo-cross-linked GelMA stably incorporated AgNPs offering a non-antibiotic bifunctional scaffold with retained antimicrobial action, while allowing ingress and attachment of mammalian cells for multiple regenerative applications. HGFs were more susceptible to the AgNPs toxicity compared with the bacterial cells. An appropriately designed 3D hydrogel construct could be used as a cytotoxicity model to better mimic the in vivo environment compared with a traditional 2D culture.

## Data Availability

The data presented in this study are available on request from the corresponding author.

## Supplementary Materials

The following supporting information can be downloaded at: https://www.mdpi.com/article/10.3390/nano13040705/s1. Figure S1: Human gingival fibroblast viable when incorporated in the three different consistencies of HyStem®-C with 200 µg/ml of AgNPs (n = 2). (A) Soft, (B) Standard, (C) Stiff. Mean ± SD; Figure S2: HyStem®-C with and without AgNPs FTIR spectra with the relevant characteristic bands labelled; Figure S3: GelMA with and without AgNPs FTIR spectra with the relevant characteristic bands labelled; Figure S4: Shows the areas were scanning electron microscopy with energy dispersive spectroscopy. B = Black, W = white. (1) HyStem®-C, (2) HyStem®-C -AgNPs, (3) GelMA, (4) GelMA -AgNPs; Table S1: HyStem®-C consistency formulation; Table S2: GelMA consistency formulation; Table S3: Silver elemental detection using EDS in HyStem®-C and GelMA with and without AgNP.
