# Supplementary material for: Three-Dimensional Evaluation of the Cytotoxicity and Antibacterial Properties of Alpha Lipoic Acid-Capped Silver Nanoparticle Constructs for Oral Applications
Source: Nanomaterials (Basel). 2023 Feb 12;13(4):705. doi: 10.3390/nano13040705 (PMC9958703; doi:10.3390/nano13040705)
Supplement: Supplementary file 1 [file nanomaterials-13-00705-s001.zip › nanomaterials-2199213-supplementary.pdf]

Supplementary file

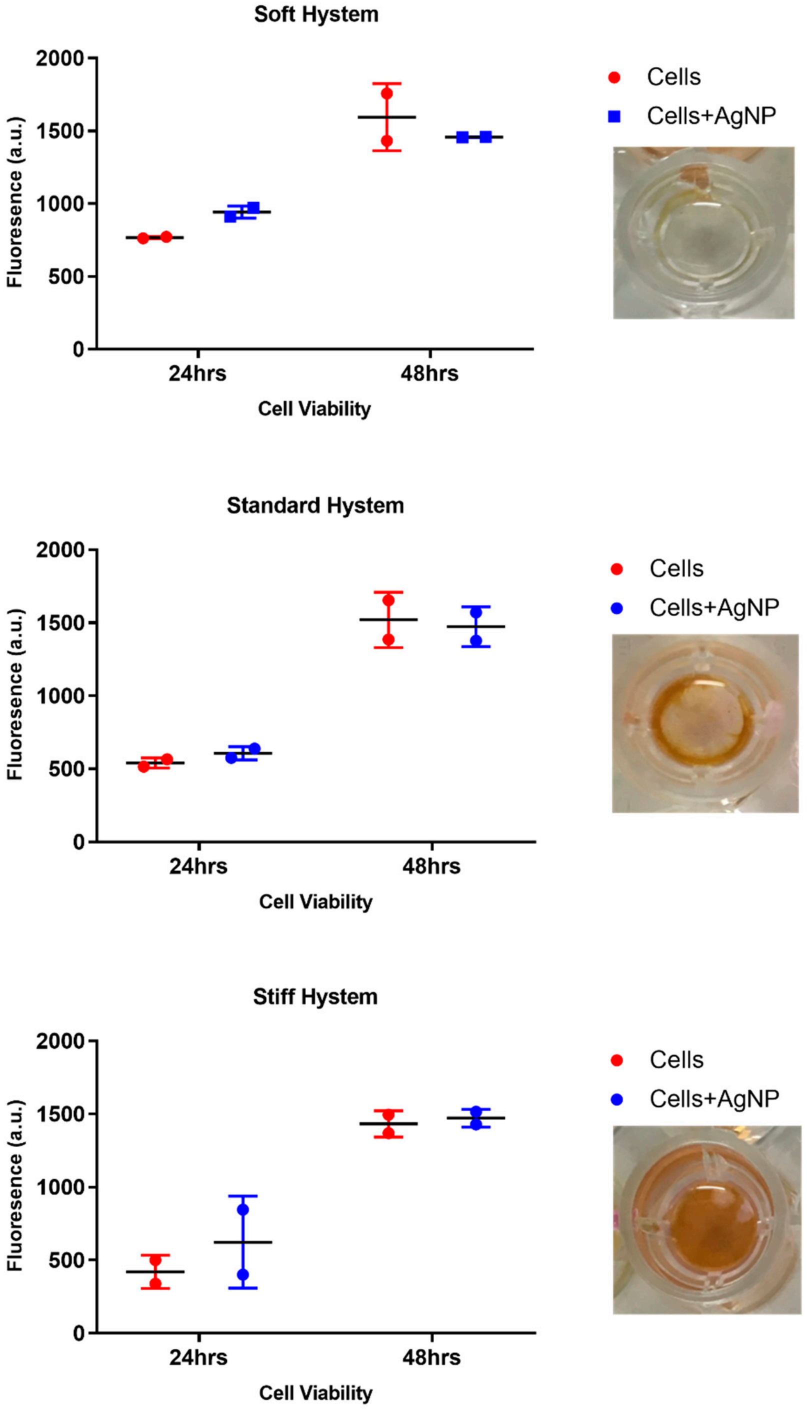

Figure S:1Human gingival fibroblast viable when incorporated in the three different consistencies of HyStem®-C with 200 µg/ml of AgNPs (n=2). (A) Soft, (B) Standard, (C) Stiff. Mean ± SD.

Table S1: HyStem®-C consistency formulation

Condition	DG-$H_2$0 (ml)	Extralink (ml)	Total (ml)
Soft	1.00	0.50	1.50
Standard	0.50	0.50	1.00
Stiff	0.25	0.50	0.75

Table S2: GelMA consistency formulation

Condition	Stock GelMA (%wt/v)	Lyophilised GelMA (mg)	PBS (µl)	Final GelMA (%wt/v)
Soft	5	50	950	2.5
Standard	10	100	900	5
Stiff	20	200	800	10

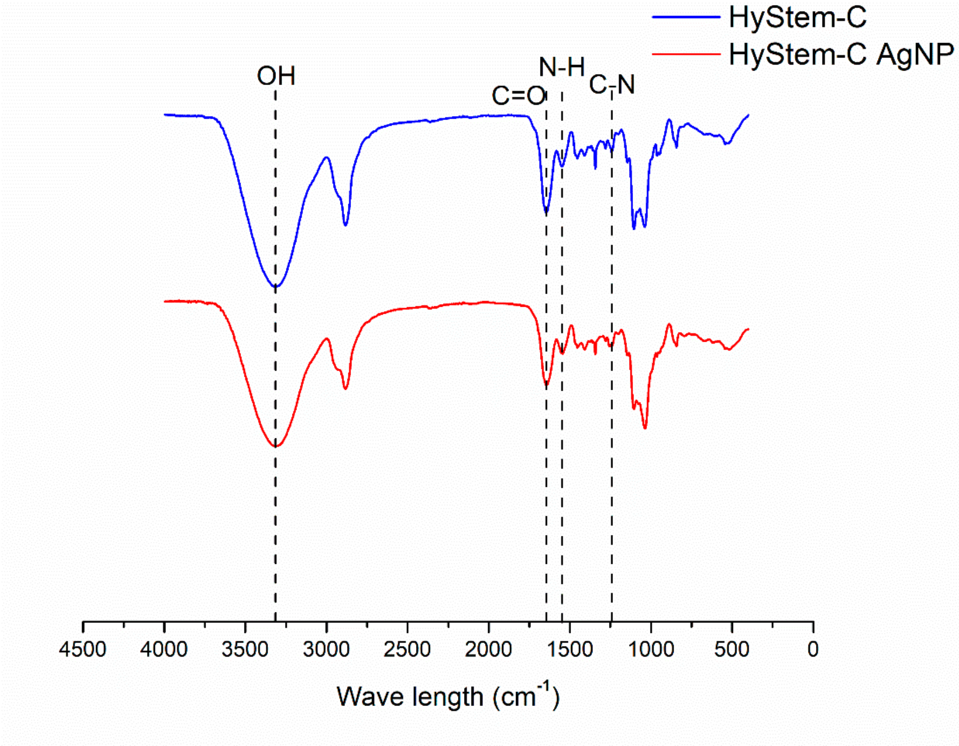

Figure S2: HyStem®-C with and without AgNPs FTIR spectra with the relevant characteristic bands labelled.

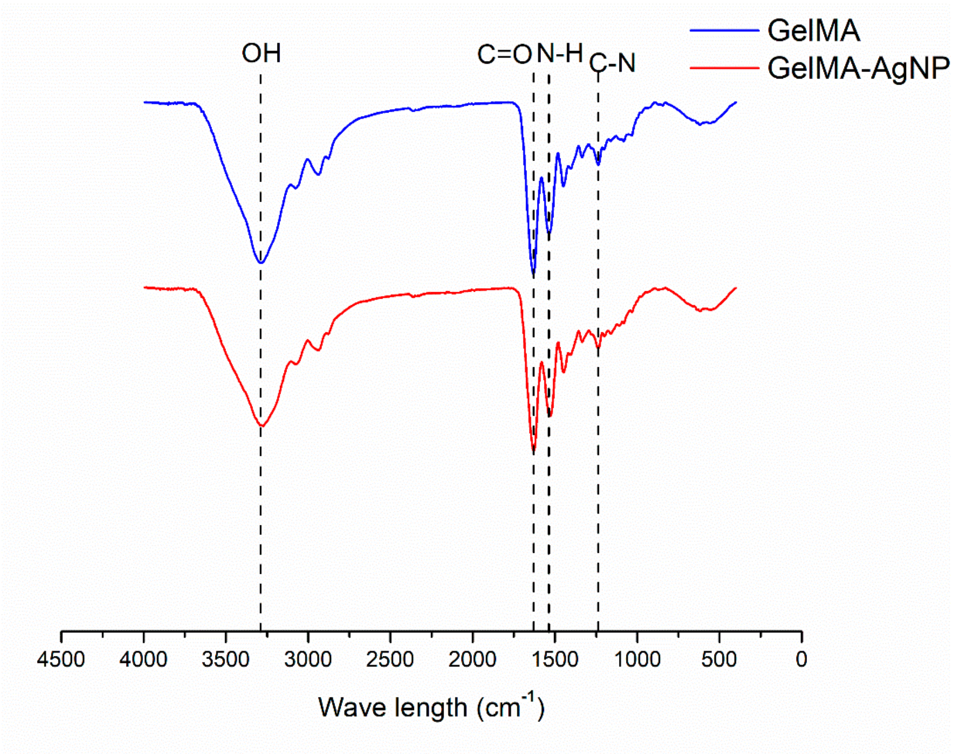

Figure S3: GelMA with and without AgNPs FTIR spectra with the relevant characteristic bands labelled.

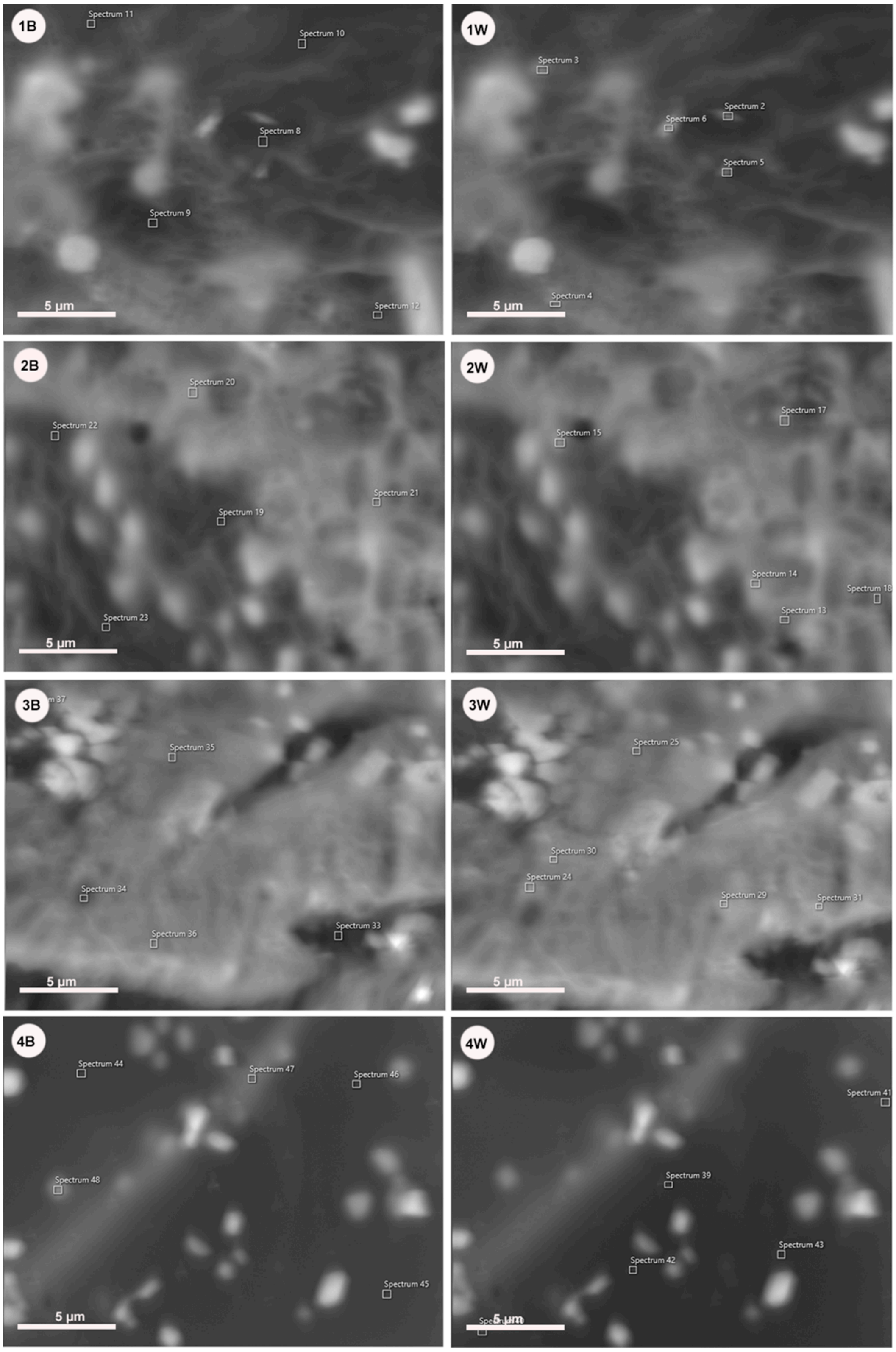

Figure S4: shows the areas were scanning electron microscopy with energy dispersive spectroscopy. B=Black, W= white. (1) HyStem®-C, (2) HyStem®-C -AgNPs, (3) GelMA, (4) GelMA -AgNPs.

Table S3: Silver elemental detection using EDS in HyStem®-C and GelMA with and without AgNP

Sample	Hydrogel	White areas					Black areas				
1	HyStem®-C	-	-	-	-	-	-	-	-	-	-
2	HyStem®-C + AgNP	+	+	+	+	+	+	+	+	+	+
3	GelMA	-	-	-	-	-	-	-	-	-	-
4	GelMA + AgNP	+	-	+	+	+	-	-	-	-	-
